# Direct fitness benefits explain mate preference, but not choice, for similarity in heterozygosity levels

**DOI:** 10.1111/ele.12827

**Published:** 2017-09-03

**Authors:** Lies Zandberg, Gerrit Gort, Kees van Oers, Camilla A. Hinde

**Affiliations:** ^1^ Behavioural Ecology Group Wageningen University and Research De Elst 1 6708 WD Wageningen The Netherlands; ^2^ Department of Animal Ecology Netherlands Institute of Ecology (NIOO‐KNAW) Droevendaalsesteeg 10 6708 PB Wageningen The Netherlands; ^3^ Department of Biometris Wageningen University and Research Droevendaalsesteeg 1 6708 PB Wageningen The Netherlands

**Keywords:** Great tit, heterozygosity, mate choice, mate preferences, relatedness, reproductive success, sexual selection

## Abstract

Under sexual selection, mate preferences can evolve for traits advertising fitness benefits. Observed mating patterns (mate choice) are often assumed to represent preference, even though they result from the interaction between preference, sampling strategy and environmental factors. Correlating fitness with mate choice instead of preference will therefore lead to confounded conclusions about the role of preference in sexual selection. Here we show that direct fitness benefits underlie mate preferences for genetic characteristics in a unique experiment on wild great tits. In repeated mate preference tests, both sexes preferred mates that had similar heterozygosity levels to themselves, and not those with which they would optimise offspring heterozygosity. In a subsequent field experiment where we cross fostered offspring, foster parents with more similar heterozygosity levels had higher reproductive success, despite the absence of assortative mating patterns. These results support the idea that selection for preference persists despite constraints on mate choice.

## Introduction

Individuals can incur fitness benefits by choosing the right mate (Andersson 1994). Therefore, preferences are expected to evolve for traits that advertise mate quality. Such preferences can be additive, or non‐additive suggesting preference for compatibility (Neff & Pitcher 2005). Selection for compatible mates predicts that individuals prefer a mate with whom they would achieve the highest reproductive success, even though this mate may not necessarily be the universally ‘best individual’ (Drickamer *et al*. 2003; Ihle *et al*. 2015). Preference functions can have different directions and shapes (Edward 2014) and can consistently differ between individuals (Brooks & Endler 2001; Forstmeier & Birkhead 2004). Only by studying the reproductive benefits individuals gain from finding a preferred mate, we can unravel the selective pressures underlying the evolution of these mate preferences (Andersson 1994).

Even though mate preferences may be at the basis of mate choice, sampling strategy and environmental or social constraints can largely influence that choice (Jennions & Petrie 1997; Wagner 1998). For instance the presence of intrasexual competition and the availability of potential mates limit the possibilities for an individual (Jennions & Petrie 1997; Wagner 1998; Bro‐Jørgensen 2002). In addition, since searching for a mate is costly, the effort invested in finding a suitable mate may be dependent on the condition of the chooser (reviewed in Cotton *et al*. 2006a). Therefore, the mate that an individual obtains (mate choice) may not represent its mate preference, as often assumed. Correlating fitness benefits with the observed choice of social partner instead of the measured preferences will therefore lead to confounded conclusions about the role of mate preferences in sexual selection. Instead, to understand fitness consequences and the evolution of mate preferences, preference and choice should be studied as two distinct processes. Although studies on mate preference, mate choice and mating patterns, and on the fitness effects of choice are abundant, there is, to our knowledge, none that has combined all three and tested what the fitness benefits of mate preferences are under mate choice constraints in a wild population.

Genetic characteristics are important for reproductive success (Foerster *et al*. 2003; Kempenaers 2007; García‐navas *et al*. 2009), and are thus expected to affect mate preference. Heterozygosity, the genetic variability within an individual, is known to be positively correlated with fitness aspects such as reproductive success, survival, immunocompetence and parasite resistance (reviewed in Kempenaers 2007 and Chapman *et al*. 2009). An individual can thus potentially increase its fitness by selecting a mate with whom it would produce heterozygous and therefore ‘fitter’ offspring (Tregenza & Wedell 2000; Tomiuk *et al*. 2007; Szulkin *et al*. 2009). By finding an unrelated, or genetically dissimilar mate, offspring heterozygosity can be increased and the negative effects of inbreeding avoided when inbreeding depression is larger than outbreeding depression (Van de Casteele *et al*. 2003; Szulkin *et al*. 2007). Moreover, although most studies focus on these indirect effects, genetic traits can have direct effects on reproductive success as well, especially in species with biparental care. For instance heterozygous females have been shown to lay larger clutches (Foerster *et al*. 2003), and heterozygous males have better territories (Seddon *et al*. 2004; Ryder *et al*. 2010), and feed their offspring more often (García‐navas *et al*. 2009).

Here we tested 139 wild great tits (*Parus major*), both males and females, for their preference for mate heterozygosity, relatedness and simulated offspring heterozygosity, and how such preferences relate to the choosers' own genetic characteristics. In a subsequent experiment, we studied how mating patterns relate to genetic characteristics. By cross fostering chicks between broods, we assessed the direct and indirect benefits of the (biological and foster) pairs' heterozygosity and relatedness on reproductive success. We aimed at investigating how mate preferences are reflected in mate choice (pair formation in the wild), and what the direct and indirect fitness benefits of these preferences are. Using this unique experiment, we were able to study the evolutionary benefits of sexual selection, by measuring mate preferences, and the reproductive benefits of these preferences under mate choice constraints in a wild population. Here we show that, despite the fact that mate preferences might not be reflected in choice, there is potential for selection on preferences for genetic advantages via direct reproductive benefits.

## Materials and methods

### Study population

We used wild great tits originating from six field sites for the preference experiments. We caught all focal birds (choosers in the preference test) in the main field site Boslust (5°85′ E, 52°01′ N), whereas the stimulus birds (chosen individuals in the preference tests) originated from more distant field sites Heijkamp (5°83′ E, 52°03′ N), Roekelse bos (5°71′ E, 52°08′ N), Westerheide (5°84′ E, 52°02′ N), Lichtenbeek (5°85′ E, 52°00′ N) and Bennekomse bos (5°69′ E, 52°00′ N) to minimise the chance they were familiar with the focal birds. Great tits pair throughout the winter period (Culina 2014), therefore we have tested birds in the months of January, February and early March, a period where they are highly likely to show mate preferences. On eight evenings in total, between January – March in 2014 and 2015 we caught 420 birds in total while they were roosting in nest boxes (on two evenings in January 2014 we caught 30 birds and we caught 60 birds on two evenings in February – March 2014 and on four evenings in January – March 2015), and transported them to the bird housing facilities at the Netherlands Institute of Ecology (NIOO‐KNAW, Wageningen, the Netherlands). At this facility, we banded and weighed them and photographed their breast stripe, after which we housed them individually in standard cages (0.9 × 0.4 × 0.5 m), with a solid bottom, top, side and rear walls, a wire‐mesh front and three perches. The birds had *ad libitum* access to water and sunflower seeds, supplemented daily with commercial egg mixture, ground peanuts, live mealworms, soldier fly larvae, dead wax moth larvae, green bottle fly larvae and crickets. Following the 6 days of preference tests, we took a blood sample (10 μL) by puncturing the brachial vein, and measured weight and tarsus length. After this we released the birds back into the field site of origin. Three birds out of the 420 birds caught died after their first night at the facilities of the NIOO‐KNAW.

### Preference tests

**Figure 1:**
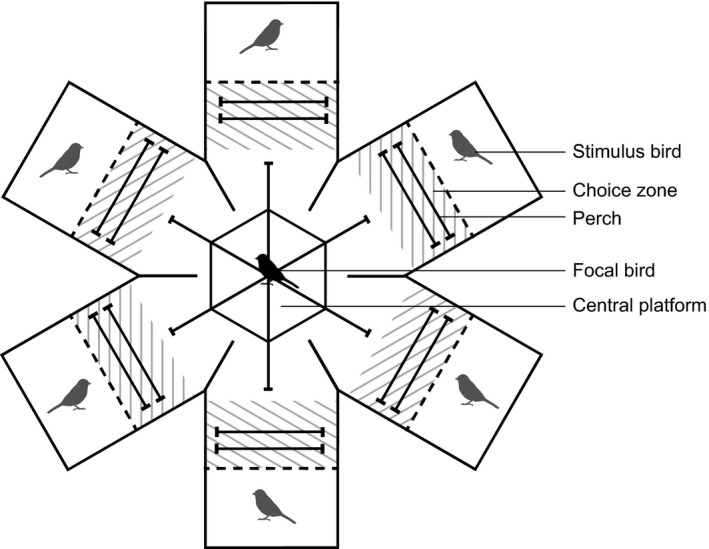
Experimental setup mate preference test. Birds were tested for their preference in a six‐choice test room. From the hexagonal central platform the focal bird could observe all stimulus birds, whereas from the perches in the choice zone only one stimulus bird was visible. Time spent in each of the choice zones was measured.

We carried out mate preference tests at NIOO‐KNAW. All tests took place in a test room (4.0 × 2.4 × 2.5 m) with white walls, and high‐frequency fluorescent lights to mimic natural lighting conditions. We tested individual mate preferences using a carrousel shaped six‐choice chamber (Fig. 1). With the six‐choice test we were able to measure individual directional and quadratic (stabilising or disruptive) preferences (Edward 2014). Great tits keep local dominance in their breeding territories throughout the winter, and often forage in larger fission‐fusion foraging flocks. Encountering six birds of the opposite sex is therefore not unrealistic for this species. For every 10 focal birds we caught 20 stimulus birds (thus in total we always caught either 30 or 60 birds per evening). Eighteen of these birds participated in the experiment and two extra birds were caught in case of health or other issues. Each focal bird was tested with three different groups of six stimulus birds each. In our final dataset we tested 69 focal females and 70 focal males for their preference with a total of 42 groups of six stimulus birds (*N* = 252).

In this six‐choice setup each focal bird could see all stimulus birds from the central perch, but when in front of a stimulus cage it could not see the other stimulus birds. The stimulus birds could not see each other (Fig. 1). For more details on the setup see Appendix S1‐A in the Supporting information.

For every test, we placed six stimulus birds in the test setup and gave them 15 min to habituate. During the test, all birds had access to a piece of apple and before each test they received five mealworms in a cup (to hide them from view for the focal bird). In most cases, the stimulus birds had eaten all the mealworms before the focal bird was introduced. When the focal bird was introduced into the setup, recording started and lasted for 45 min. We tested each focal bird with three groups of different stimulus birds (groups A, B and C), of which one group was repeated (order of testing: ABCA). With this repeated test we tested the overall repeatability of preference for focal‐stimulus combinations by conducting a one‐way anova with focal‐stimulus combination as a factor and association time as the dependent variable. Repeatability was calculated using the mean squares within and between focal‐stimulus combinations (*r *= 0.10 ± 0.04). For further analysis we only used the second repeated test on A to have equal numbers of tests for every focal‐stimulus dyad and avoid habituation effects of the first test. Every stimulus bird participated in 13 or 14 trials with 10 focal birds, divided over four mornings and afternoons over a period of 6 days. We randomised the positions of the stimulus birds within group. Every group of stimulus birds was composed blind with regard to the natural variation in heterozygosity and relatedness to the focal bird. From the videos, we calculated the time spent in front of each stimulus bird, which is commonly used as a measure of preference. Great tits do not show clear courtship or copulation solicitation behaviours outside the fertile period and it is therefore difficult to validate these tests and the association time measure using behavioural data. However, in numerous species association time in a choice test has been shown to predict courtship behaviours or pair formation (Clayton 1990; Witte 2006; Lehtonen & Lindström 2008; Jeswiet & Godin 2011) and to correlate with reproductive success (Drickamer *et al*. 2003). For our analysis, we modelled the proportion of association time, i.e. the association time with each stimulus bird divided by the total association time that this bird spent with all stimulus birds. Using this measure we were able to separate the directional preference for traits from variation in the motivation to choose (the total time spent in all choice zones) (Cotton *et al*. 2006b). Trials in which the focal bird did not visit any of the stimulus birds (*N* = 53) were excluded from the analysis.

### Reproductive success

During the breeding seasons following the mate preference tests, we monitored all breeding attempts in the Boslust population, from where the focal individuals originated. Boslust is a mixed wood forest of *c*. 70 ha containing 130 nest boxes. During the breeding season we checked unoccupied nest boxes every 5 days for nesting activity. For every nest we estimated the egg‐laying date, start of incubation, hatching date and fledging date and recorded the clutch size, brood size and the number of fledged offspring (as in Hinde 2006). We weighed the chicks on the day of hatching (day 0), and gave them a down code by selectively removing down feathers to individually identify each chick within a brood. These down codes were visible until at least day 6, after which we gave them an uniquely numbered aluminium ring. On day 1 after hatching we cross fostered the chicks with chicks from two other broods that had hatched on the same day, matched for weight as in Brinkhof *et al*. (1999) in such a way that all chicks were raised by foster parents. In 2014 and 2015 we cross fostered 26 and 48 broods, with 191 and 332 chicks respectively. On day 3 after hatching we took a blood sample (3 μL) from all chicks from the metatarsal vein. Chicks were weighed 14 days after hatching (to the nearest 0.01 g using a digital scale) as a measure of fledging weight, which is closely correlated with first year survival (Van Balen 1973), and we measured their tarsus length (to the nearest 0.1 mm using callipers). When the nestlings were 10 days old we caught the adults at the nest using spring traps. We recorded adult body mass, tarsus length and took a blood sample (10 μL) from the brachial vein. We fitted unbanded adults with an aluminium numbered ring. Each nest was checked after 21–25 days for chick‐fledging.

### Genotyping and genetic traits

We genotyped all birds that participated in the mate preference experiments (344 individuals) and the breeding season (142 adults, 486 chicks) across 20 polymorphic microsatellite markers. Using known mother‐offspring dyads we detected the occurrence of null alleles and other irregularities. On the basis of this analysis we excluded three microsatellite loci from further analyses because of the non‐reliability of their results. Full details on marker‐characteristics and the genetic analyses can be found in Appendix S1‐B.

We used homozygosity by locus (Aparicio *et al*. 2006) to estimate individual genetic diversity and calculated this measure for all genotyped individuals with the R package GENHET (Coulon 2010). Because the HL index represents homozygosity instead of heterozygosity we transformed the HL values into an estimate of heterozygosity by calculating the complement of HL (1‐HL). Using the package Rhh (Alho *et al*. 2010) we determined that our sample of 17 microsatellites is very likely to be representative of genome wide heterozygosity. See Appendix S1‐B for more details on the test of genome wide heterozygosity and the choice for HL as a measure of heterozygosity. For each focal‐stimulus dyad in the mate preference tests, we simulated offspring heterozygosity using the program STORM (Frasier 2008). For this the average HL estimate of 100 simulated offspring was calculated over 1000 iterations and averaged for each dyad (*N* = 2046).

We estimated marker‐based relatedness by calculating the pairwise *r* following the method of Wang (2002) in the program Coancestry (Wang 2011), as this measure fitted our social pedigree best (see Appendix S1‐B). The relatedness values range from −1 to 1, in which values of 0 represent random allele sharing, and positive and negative values, respectively, represent more and less sharing than at random, based on the allele frequencies in the population.

Using the microsatellite data from 17 loci, we assigned paternity of offspring using Cervus 3.07 (Marshall *et al*. 1998). For 49 offspring, in 23 broods, the social father was unlikely to be the sire. By comparing these offspring with all other males, we were able to identify the extra‐pair father for 15 offspring (see Appendix S1‐B).

### Statistical analyses

#### Mate preference

To analyse the proportion of time spent associating with each stimulus bird we used a binomial generalised linear mixed model with a logit link function. The fixed part of the model contained as explanatory variables: heterozygosity of focal and of stimulus birds; relatedness, squared relatedness and offspring heterozygosity for each focal and stimulus dyad; sex of the focal bird; interaction of focal and stimulus heterozygosities, of focal bird's heterozygosity and relatedness and of sex and aforementioned variables. The random part of the model contained different components, following the experimental set‐up as closely as possible. In case of a significant interaction of continuous variables, we used as a check on possible shortcomings of the systematic part of the model categorised versions of the variables: we grouped the values of a variable into three groups, with cutpoints based upon tertiles, and formulated the model using these factors, thereby allowing a more flexible response surface. These extra analyses confirm the general patterns that we found in the interactions of the continuous variables, we therefore only report these in the supplementary information. For details on the analysis and results we refer to Appendix S1‐C and S3‐D respectively.

We started the analysis with an omnibus test on the full model. Since this was significant (*F* = 1.95, *P* = 0.03) we proceeded with stepwise backward selection to obtain the minimal adequate models. Non‐significant terms were deleted stepwise, starting with the highest order interactions and/or the least significant term (*P* < 0.05). Because of the high number of variables in the model and the possibility of false positives we treated *P*‐values between 0.05 and 0.01 with more caution and refrain from drawing strong conclusions. Because of this we will describe these results in more detail in Appendix S4. The full model containing all variables of interest is included in Appendix S5.

#### Assortative mating

We tested the null hypothesis of random pairing between males and females by performing a permutation test in which we compared the test statistic, that is, the correlation between the males and females of a pair, to a sampling distribution generated by randomly permuting the females and computing the correlation between randomly paired couples (10 000 permutations).

We explored whether individuals paired differently from random with regard to relatedness. To test this we compared the observed distribution of relatedness between mates, with a distribution obtained if individuals mated randomly with regard to relatedness in R (version 2.3.2, R Development Core Team 2015). We generated randomly mated pairs (within each year) with 100 000 iterations and compared the confidence interval of the average relatedness between these randomly generated pairs with the average relatedness values in the observed population. The birds could have been restricted in their choice due to the local availability of potential mates; hence, we also ran an analysis accounting for spatial structure (see Appendix S3‐B). Furthermore, to check whether catching birds for preference testing might have influenced mate choice patterns we also compared mating analyses between tested and untested pairs (see Appendix S3‐C). These additional analyses did not change our results and therefore we present only the uncorrected analyses.

#### Reproductive success

We tested the effects of parent heterozygosity and relatedness on fledging weight (weight of the chicks when 14 days old), of both biological and foster parents. For this we ran a linear mixed model using the R package *lme4* version (version 1.1‐12, Bates *et al*. 2015), with biological brood and foster brood as random effects to account for the cross‐foster design and the multiple chicks per brood. As explanatory variables we added heterozygosity of both parents of the biological and the foster parent pairs and the relatedness and square of relatedness between the pair members. For effects of biological fathers and relatedness we used the genetic sire, which in some cases was the extra‐pair sire. We also tested for an interaction effect of parental heterozygosity on reproductive success. In addition, to test whether the effect of the relatedness of the partner depended on the heterozygosity of the individual, we also tested the interaction between relatedness and heterozygosity for both biological and foster parents. Moreover, we also tested whether chick heterozygosity influenced weight by adding this as an explanatory variable to the model. Because brood size and offspring sex (as categorical variable) are known to affect chick weight we also controlled for these. We also added brood size, catch date, year (as a categorical variable) and the interaction between catch date and year (catch date*year) as control variables. We started the analysis with an omnibus test on the full model. Since this was found to be significant ($X_{20}^{2}=43.55$, *P* = 0.002) we proceeded with stepwise backward selection to obtain the minimal adequate models.

We tested for differences in fledging probability for each chick with a binary generalised linear mixed model using the R package *lme4* (Bates *et al*. 2015), with fledging (yes or no) as a dependent variable and heterozygosity, relatedness for biological and foster parents as explanatory variables. We also tested for an interaction effect of parental heterozygosity. To test whether relatedness effects differed depending on the heterozygosity of the parents we included the interaction between relatedness and heterozygosity for biological and foster parents. We added brood size, hatch date, offspring sex and year as control variables (offspring sex and year as a categorical variables). Biological brood and foster brood were added as random effects to account for the cross‐foster design. Similar to the preference models we started the analysis with an omnibus test on the full model. Since this was found to be significant ($X_{13}^{2}=29.34$, *P* = 0.006) we proceeded with stepwise backward selection to obtain the minimal adequate models. In case of significant interactions we checked the systematic part of the model using categorised versions of the variables (see Appendix S3‐D) and we treated *P*‐values between 0.05 and 0.01 with more caution. These results are described in more detail in Appendix S4. Full models containing all variables of interest are included in Appendix S5.

## Results

### Mate preferences

**Figure 2:**
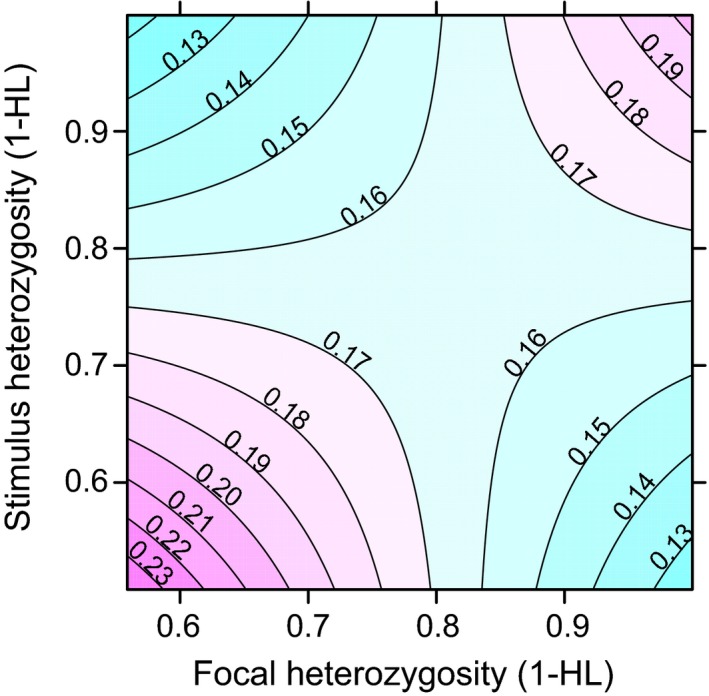
Preferences for heterozygosity levels. Individuals spent more time with stimulus birds with similar heterozygosity levels. Association time with each stimulus bird was calculated as the fraction of the total time spent with all stimulus birds. Red colours indicate that relatively more time was spent and green colours indicate that relatively less time was spent.

Individuals did not spend more time with stimulus birds with which they could theoretically optimise offspring heterozygosity (HL_offspring_, GLMM: *F*_1,66.3_ = −0.24, *P* = 0.81, Table 1). Time spent with a stimulus bird was influenced by an interaction between the chooser's and stimulus birds' heterozygosity (Fig. 2; HL_focal_ × HL_stimulus_, GLMM: *F*_1,62.93_ = 2.76, *P* = 0.008, Table 1). Very heterozygous birds spent more time with heterozygous birds, and vice versa, homozygous birds spent more time with homozygous birds.

**Table 1:** Mate preferences – minimal adequate model

	Estimate	Num d.f.	Denom d.f.	Test statistic	*P* value
Minimal adequate model					
Intercept	−1.63	1	66.46	−47.52	< 0.0001
HL_focal_	0.14	1	204.90	2.47	*0.01*
HL_stimulus_	0.03	1	160.20	0.05	0.96
HL_focal_ × HL_stimulus_	6.76	1	62.93	2.76	**0.008**
Relatedness	0.30	1	63.05	1.65	0.10
Relatedness²	−2.33	1	274.20	−2.39	*0.02*
Relatedness × HL_stimulus_	−3.10	1	277.10	−2.06	*0.04*
Dropped terms					
HL_offspring_	−0.13	1	66.30	−0.24	0.81
Sex (female)	0.02	1	61.65	0.23	0.82
HL_stimulus_ × sex (female)	0.95	1	164.80	0.93	0.35
HL_focal_ × HL_stimulus_ × sex (female)	5.67	1	61.06	1.05	0.30
HL_offspring_ × sex (female)	0.90	1	43.50	0.94	0.35
Relatedness × HL_focal_	−1.42	1	63.37	−0.66	0.51
Relatedness × sex (female)	−0.29	1	62.97	−0.68	0.50
Relatedness² × sex (female)	1.55	1	282.00	0.71	0.48

Table consists of all factors tested in the binomial mixed model with proportion of time spent with each of the stimulus birds as the dependent variable (*N*_focals_ = 116, *N*_tests_ = 359). Given is the estimate, the degrees of freedom (d.f.), the test statistic (*F*‐value) and the significance (*P*‐value). Significant terms (P<0.01) are indicated in bold, and marginally significant terms (P= 0.05 ‐ 0.01) are indicated in italics (and described in Appendix S4). A random effect for stimulus bird identity (mean ± SE; 0.22 ± 0.04) *r* and random slopes for focal bird identity with respect to the stimulus bird's heterozygosity (2.41 ± 0.70), relatedness (1.46 ± 0.47) and offspring heterozygosity (3.23 ± 1.98), and a random effect for test number (to allow for negative correlations among association times within one six‐choice test; (−13.89 ± 0.52) and an extra scale parameter on the original scale, were included in the model. Using backwards elimination of factors, the *P*‐values, d.f. and test statistics given come from the last model in which the factor or interaction was included. Degrees of freedom for *F*‐ and *t*‐tests were calculated using the degree of freedom approximation proposed by Kenward and Roger (1997).

### Assortative mating

Birds did not mate assortatively for heterozygosity. There was no difference between heterozygosity correlations within pairs and a randomly generated distribution of pairs drawn from the population (random correlation 95% confidence interval = [−0.23, 0.25]; correlation breeding pairs = −0.07; *N* = 70 breeding pairs; 10 000 permutations).

Individuals did not mate differently from random with regard to relatedness. The randomisation test showed no difference between relatedness within the breeding pairs and randomly generated pairs drawn from the population (random relatedness 95% confidence interval = [−0.026, 0.022]; average relatedness breeding pairs = 0.009; *N* = 70 breeding pairs; 100 000 iterations). For the complete observed and simulated distribution of relatedness and heterozygosity see Appendix S3‐A.

### Reproductive success

**Figure 3:**
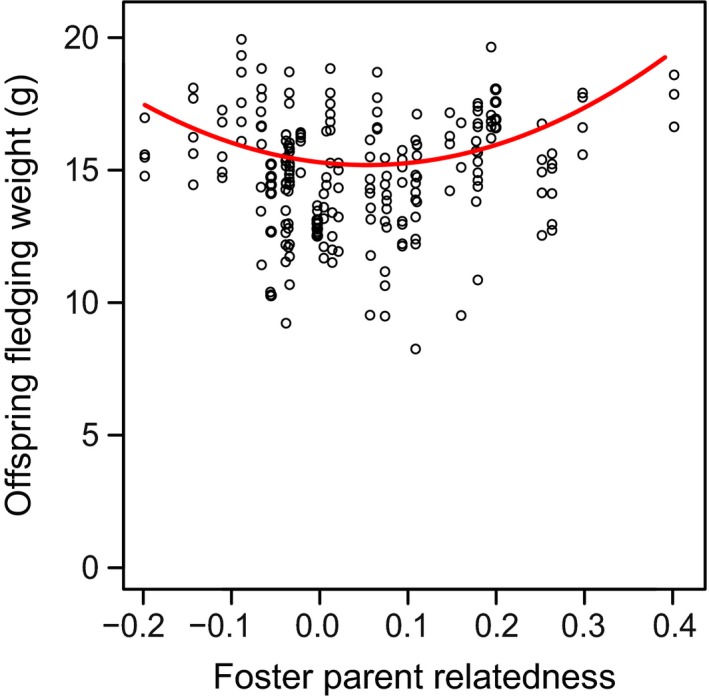
Effects of foster parent relatedness on offspring fledging weight. Offspring from very related or very unrelated foster parents had a higher fledging weight than offspring from moderately related parents.

The relatedness of foster parents showed a quadratic relationship with fledging weight (on day 14); very unrelated and very related foster parents reared heavier offspring (Fig. 3; relatedness^2^, LMM: *t*_34.91_ = 3.19, *P* = 0.003, Table S2).

**Figure 4:**
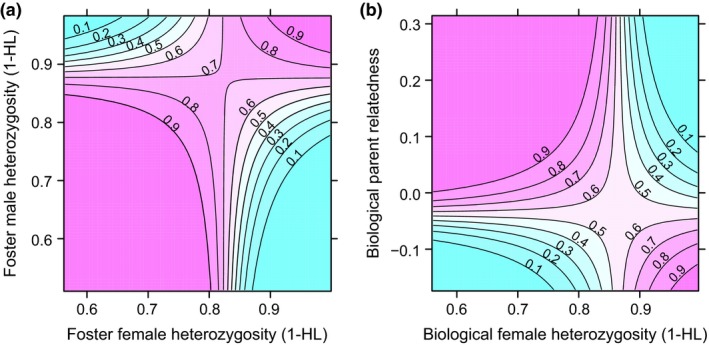
Effects of genetic characteristics on fledging success of chicks. (a) Offspring from foster parents with similar heterozygosity levels had a higher chance of fledging. (b) Fledging probability depended on the heterozygosity of the biological mother and the biological parents' relatedness. Red colours indicate a higher fledging probability and green colours indicate a lower fledging probability.

Heterozygous offspring were not more likely to fledge than homozygous offspring (HL_offspring_, GLMM: *Z* = 0.28, *P* = 0.78, Table 2). However, offspring from foster parents with similar levels of heterozygosity had a higher chance of fledging than offspring from dissimilar foster parents (Fig. 4a; HL_foster female_ × HL_foster male_, GLMM: *Z* = 3.26, *P* < 0.001, Table 2). Biological parents produce offspring with higher fledging success when they are more related and the female is more homozygous (Fig. 4b; HL_biological female_ × biological relatedness, GLMM: *Z* = −3.31, *P* < 0.001, Table 2).

**Table 2:** Offspring fledging probability – minimal adequate model

	Estimate	Test statistic	*P* value
Minimal adequate model			
Intercept	0.45	0.44	0.66
HL_foster female_	−10.02	−1.96	*0.05*
HL_foster male_	3.44	0.83	0.40
HL_foster female_ × HL_foster male_	176.96	3.26	**< 0.001**
HL_biological female_	−6.28	−1.51	0.13
HL_biological male_	−5.15	−2.07	*0.04*
Biological relatedness	5.39	1.89	0.06
HL_biological female_ × biological relatedness	−169.41	−3.31	**< 0.001**
Offspring sex (male)	−0.33	−0.86	0.39
Brood size	−0.30	−1.03	0.31
Hatch date	0.21	1.27	0.20
Year (2015)	−0.01	−0.01	0.99
Dropped terms			
HL_offspring_	0.66	0.28	0.78
Foster relatedness	5.48	1.46	0.14
HL_foster female_ × Foster relatedness	33.95	0.78	0.43
HL_foster male_ × Foster relatedness	16.83	0.37	0.71
HL_biological female_ × HL_biological male_	−3.08	−0.08	0.93
HL_biological male_ × biological relatedness	−19.18	−0.70	0.48

Table consists of all factors tested in the binary mixed model with the fledging probability of the offspring (0/1) as the dependent variable (*N* = 272). Random effects for biological brood (var ± SD: 0.00 ± 0.00) and foster brood (3.63 ± 1.90) were included in the model. Given is the estimate, the degrees of freedom (d.f.), the test statistic (*Z*‐value) and the significance (*P*‐value). Significant terms (P<0.01) are indicated in bold, and marginally significant terms (P= 0.05 ‐ 0.01) are indicated in italics (and described in Appendix S4). Biological and foster brood identity were included as random factors. Using backwards elimination of factors, the *P*‐ values, d.f. and test statistics given come from the last model in which the factor or interaction was included.

## Discussion

Despite great interest in the evolution of mating preferences and numerous studies of sexual selection processes, few actually test how selection acts on mating preferences in natural populations. Here we show that both males and females preferred potential mates with similar levels of heterozygosity to their own. While we did not find these mate preferences reflected in the observed mating patterns, individuals gained direct fitness benefits (greater fledging success) from mating assortatively for heterozygosity. These direct benefits indicate the potential for selection on preferences for genetic benefits.

Against our expectations, we did not find any mating patterns with regard to heterozygosity or relatedness. This suggests that, despite their preferences, great tits, in this study, do not all obtain a partner according to their preference. Mating patterns can differ from preferences due to different factors (Wagner 1998). The great tit is a territorial species with biparental care. Therefore, individuals become unavailable after mating and the number of available mates at any time is limited. As they have to choose from what is available at that place and time, their choice may be limited and they might have to settle with a less preferred partner. Despite this, they may still choose according to their preference from this limited pool of available mates. Moreover, compared to the sample sizes in the mate preference tests, we have only a limited dataset on mate choice. Possibly, because of these two reasons, we were not able to pick up on mating patterns in the data. Moreover, we also checked if mating patterns of birds used in the preference tests could have been affected by catching them for these tests, however this did not seem to be the case (see Appendix S3). The difference between preference and choice that we found here illustrates the importance of considering them as two distinct processes, both of which should be studied independently. By measuring preferences and the fitness aspects correlated with it, it is possible to show how selection works on these preferences (Jennions & Petrie 1997). We strongly advise that the terminology for mate preference (functions) and mate choice should be used consistently, to be able to compare studies and draw conclusions (Jennions & Petrie 1997; Wagner 1998; Edward 2014).

We expected individuals to prefer heterozygous partners, as heterozygous parents may produce more heterozygous offspring (Mitton *et al*. 1993; Hoffman *et al*. 2007), and have been shown to invest more in their offspring (Foerster *et al*. 2003; García‐navas *et al*. 2009). In our preference experiments, however, we found that rather than having a uniform preference for the most heterozygous mate (Kempenaers 2007), individuals showed a strong preference to spend time with individuals that had similar heterozygosity levels to themselves. A number of studies have found similar assortative mating patterns for heterozygosity (Bonneaud *et al*. 2006; García‐navas *et al*. 2009). Under the assumption that all individuals have the same preference for heterozygous mates, the authors suggest that assortative mating is due to competition over mates. In our preference experiments however, this pattern represents the actual preference, not choice, for similarity in heterozygosity. This preference is likely to represent a behavioural, rather than a genetic compatibility, since it benefitted the chicks through an effect of foster parents. One potential explanation for this surprising pattern may be that, assuming that heterozygosity is a trait indicating quality, assortative preferences have evolved to save low‐quality individuals energy and time in searching and to lower the risks of being abandoned by their mate for a higher quality individual (Burley 1988; Johnstone 1997; Mcnamara *et al*. 1999; Holveck & Riebel 2010). Alternatively individuals with similar heterozygosity levels may have a higher behavioural compatibility resulting in direct benefits of having this preference.”

The consequences for this preference were reflected in fledging success, as chicks raised by pairs with similar heterozygosity levels had a higher chance of fledging. This effect was not due to pre‐hatching effects such as genetic and early maternal effects, but a post‐hatching rearing effect from the foster parents. From our data we cannot determine through which mechanism these assortative pairs were more successful in rearing offspring. However, similar to our results, offspring mortality during the rearing period in zebra finches (*Taeniopygia guttata)* depended on the compatibility of the foster parents (Ihle *et al*. 2015). Possibly individuals invested more when they were able to obtain a mate they preferred, regardless of its apparent heterozygosity or quality (Drickamer *et al*. 2003; Ihle *et al*. 2015).

Against our expectations, we did not find individuals to prefer a mate with which they would produce more heterozygous offspring, which have a higher expected fitness prospect (Kempenaers 2007). Moreover, during the breeding season, we did not observe any pairing patterns based on a choice for relatedness levels. Although inbreeding has been shown to have negative effects on fitness in great tits (Szulkin *et al*. 2007), no preference or avoidance of inbreeding in mating patterns was observed in the same population (Szulkin *et al*. 2009), which is similar to the patterns we found in our population. Nevertheless, we did find a direct effect of relatedness, which is not associated with optimal in‐ or outbreeding, but an effect of the relatedness levels of the foster parents that the chicks are reared by. The offspring raised by moderately related parents are lighter, suggesting lower provisioning efforts and reproductive investment than in very related or very unrelated pairs. Which exact mechanism could underlie this higher reproductive investment for very related and unrelated pairs cannot be concluded from this dataset. Possibly the higher investment in these cases occurs because of different causes; when the pair is very related there are inclusive fitness benefits to be gained (Wang 2011) and when the pair is unrelated, and therefore genetically compatible, they invest more in potentially higher quality offspring (Burley 1988; Mays & Hill 2004).

In addition, the effect of relatedness on fledging probability depended on the female's heterozygosity levels. Although we hypothesised that especially homozygous individuals would obtain genetic benefits from finding an unrelated partner to increase their offspring heterozygosity, when biological mothers were homozygous, chicks had a higher fledging probability when she mated with a related male. These effects of the biological parents were surprising, especially since we expected the genetic characteristics to have a positive effect on offspring performance through offspring heterozygosity. Possibly these fitness effects of heterozygosity will only become apparent later in life (Szulkin *et al*. 2007). Apart from genetic effects the higher fledging success might also work through other pre‐hatching effects that may be related to male attractiveness, such as maternal investment in egg size (Horváthová *et al*. 2012), yolk carotenoids (Marri & Richner 2014) or yolk androgens (Kingma *et al*. 2009).

Our results highlight the importance of testing not just for the indirect but also for the direct benefits of choice for genetic traits. For species with biparental care, most studies correlate parental heterozygosity or relatedness with different aspects of reproductive success, such as growth, provisioning or fledging success. Often, when no direct benefits can be observed, genetic effects are assumed by default (Ryder *et al*. 2010). By using a cross‐foster design these direct and indirect effects can be teased apart to elucidate why individuals show particular mate preferences and what the benefits are of these preferences.

To conclude, in contrast to what is commonly assumed, we found that individuals do not show mate preferences that optimise offspring heterozygosity, but individuals prefer a mate with similar heterozygosity levels. These preferences for heterozygosity similarity can be explained by direct fitness benefits, indicating the importance of considering both indirect and direct benefits and effects of behavioural compatibility when studying mate choice for genetic traits. Moreover, our findings highlight the importance of studying preference and choice as two distinct processes, to further understand the selection pressures working on preferences. In addition, despite the fact that in natural situations mating patterns are not always determined by preference alone, we indicate the potential for selection for preferences for genetic characteristics via reproductive benefits.

## Author contributions

LZ, CAH and KO designed the study, LZ collected the data, LZ and GG analysed the data and LZ drafted the initial version of the manuscript. All authors contributed to later versions of the manuscript.

## Authorship

LZ, CAH and KO designed the study, LZ collected the data, LZ and GG analysed the data and LZ drafted the initial version of the manuscript. All authors contributed to later versions of the manuscript.

## Data accessibility statement

Data available from Figshare online digital repository: https://doi.org/10.6084/m9.figshare.5286091
