# Supplementary material for: Direct fitness benefits explain mate preference, but not choice, for similarity in heterozygosity levels
Source: Ecol Lett. 2017 Sep 3;20(10):1306–14. doi: 10.1111/ele.12827 (PMC5639373; doi:10.1111/ele.12827)
Supplement: Supplementary file 1 [file ELE-20-1306-s001.pdf]

SUPPLEMENTARY INFORMATION

Direct fitness benefits explain mate preference, but not choice, for similarity in heterozygosity levels - *Lies Zandberg, Gerrit Gort, Kees van Oers, Camilla A. Hinde*

Appendix S1: Methods

A. Detailed description of the mate choice setup

The mate choice setup consisted of a central zone for the focal bird (diameter: 1.48 m, height: 1.10 m) and 6 stimulus cages placed around the central zone (0.5 x 0.3 x 0.5 m). The stimulus cages were separated from the central area by UV-transmitting transparent PMMA panels, to allow visual contact between the focal bird and a stimulus bird. All birds were within auditory range of each other. The ceiling of both the focal compartment and of the stimulus cages was also made of UV transmitting PMMA. In the middle of the central focal compartment we placed a hexagonal platform (0.4 m high) to prevent visual contact between the stimulus birds. On top of this we placed a perch with 6 branches of equal length in the direction of the stimulus cages, from which the focal bird could observe all stimulus birds. Two perches were present in front of every stimulus cage for the focal bird to sit on in proximity of the stimulus bird. From this position it could only observe that specific stimulus bird. The movements of the focal bird were recorded using a central camera pointing down from the ceiling (Panasonic WV-CP500). Using EthoVision XT software (Noldus Information Technology) we analysed the videos to calculate for every test the time that the focal bird spent in each choice zone.

B. Genetic analyses and microsatellite markers

DNA was isolated from blood samples using the 96-well Genomic DNA Kit following the manufacturer's instruction (FAVORGEN Biotech Corporation, Taiwan) and was quantified using a Nanodrop (Thermo Fisher Scientific). We genotyped all birds that participated in the mate preference experiments (344 individuals), and the breeding season (142 adults, of which 86 had been in the mate choice experiment, and 426 chicks) across 20 polymorphic microsatellite markers. Blood samples of 9 birds tested in the mate preference tests were missing and thus omitted from the genotype analysis. In the breeding season 37 chicks died

before a blood sample could be taken, and were therefore not genotyped. Using known mother-offspring dyads it was possible to detect the occurrence of null alleles and other irregularities. On the basis of this analysis the following three microsatellite loci were excluded from further analyses because of the non-reliability of their results: PmaD130, PmaGAn40 and Pma196 (Kawano 2003; Saladin *et al.* 2003). See table S1 for the properties of the markers used in this study.

Table S1. Summary of properties of microsatellite markers used in this study. *The table shows the following information: range of allele sizes, category (neutral or functional), number of alleles (K), expected heterozygosity (H_{Exp}), observed heterozygosity (H_{Obs}), deviations from Hardy-Weinberg Equilibrium (HW), with NS = not significant and ND = not done, and reference for each locus. Summary is based on samples from all samples from adult birds (N=486) analysed using CERVUS (Marshall et al. 1998).*

Locus	Allele sizes	k	H_{Exp}	H_{Obs}	HW	Reference
PmaC25	310-340	11	0.862	0.879	NS	Saladin *et al.* 2003
PmaD105	375-427	14	0.835	0.841	NS	Saladin *et al.* 2003
PmaGAn27	196-266	25	0.922	0.904	NS	Saladin *et al.* 2003
PmaTAGAn71	171-214	12	0.808	0.812	NS	Saladin *et al.* 2003
PmaTGAn33	249-331	21	0.897	0.901	NS	Saladin *et al.* 2003
PmaCAn1	112-143	14	0.836	0.800	NS	Saladin *et al.* 2003
Pma303	148-153	2	0.165	0.165	ND	Kawano 2003
PmaTGAn64	301-323	7	0.390	0.400	NS	Saladin & Richner 2012
PmaD22	386-456	18	0.900	0.868	NS	Saladin *et al.* 2003
PmaTGAn59	85-144	21	0.915	0.905	NS	Saladin & Richner 2012
Titgata87	161-311	39	0.891	0.837	NS	Wang *et al.* 2005
PmaTAGAn73	212-251	11	0.811	0.834	NS	Saladin & Richner 2012
PmaCAn2	99-160	27	0.907	0.901	NS	Saladin & Richner 2012
PmaTAGAn86	141-223	23	0.866	0.821	NS	Saladin *et al.* 2003
Pma69u	218-244	11	0.723	0.705	NS	Kawano 2003
PmaTGAn54	345-452	31	0.872	0.824	NS	Saladin & Richner 2012
Titgata84	554-584	9	0.549	0.564	NS	Wang *et al.* 2005

Extrapair paternity

Using the microsatellite data from 17 loci, paternity of chicks was assigned using a likelihood approach in the software program Cervus 3.07 (Marshall et al. 1998). These loci had a combined second-parent exclusion probability (Pre) of 0.9999999945. We calculated critical values of LOD (log likelihood ratio) and delta (difference in LOD scores between the most likely candidate parent and the second most likely candidate parent) using the following parameters in CERVUS: 10000 cycles, 98% of loci typed, error rate 0.01%, two candidate parents. Offspring were assigned to be extra-pair when these critical values were exceeded in the comparison of the genotypes of the mother, the putative father and the offspring. 49 offspring of in total 23 broods were classified to be sired by an extra-pair father. By comparing these offspring genotypes with all known males from this field site in our dataset, we were able to identify the extra-pair father for 15 offspring.

Heterozygosity

Using the R-package '*Inbreedr*' (Stoffel *et al.* 2016) we tested whether our sample of 17 microsatellites could be used as a measure of genome-wide heterozygosity by calculating the heterozygosity-heterozygosity correlation (HHC) and the g_2 estimator of identity disequilibrium (Balloux *et al.* 2004; David *et al.* 2007; Stoffel *et al.* 2016). For this analysis we used the genotypes from all adult birds (N=486) (Balloux *et al.* 2004). Adding chicks in this analysis would overestimate the presence of rare alleles and with it heterozygosity for these alleles, causing a lower heterozygosity-heterozygosity correlation. Correlating heterozygosity based on one half of the markers with the heterozygosity of the other half of the markers gave a mean correlation of r = 0.08, 95% CI = 0.004 - 0.148 (1000 iterations). Moreover, the g_2 (David *et al.* 2007) for this dataset differed significantly from zero g2 = 0.0019, P = 0.04 (1000 iterations, and 1000 permutations). Thus, together, the HHC and g_2 indicate that marker heterozygosity is representative of genome-wide heterozygosity in this study system.

As a measure for heterozygosity we calculated homozygosity by locus (HL; Aparicio *et al.* 2006). HL accounts for allele frequencies and rare alleles, which makes it particularly suitable

for populations with high immigration rates, and a high expected heterozygosity (above 0.4-0.6) (Aparicio *et al.* 2006). As with this population and this set of alleles the expected heterozygosity lies around 0.77, the HL as a measure for heterozygosity is very appropriate. We therefore decided to use HL as a measure of heterozygosity. With this measure, birds that are completely homozygous have values of 1, and completely heterozygous individuals would have a value of 0. Because the HL index represents homozygosity instead of heterozygosity we transformed the HL values into an estimate of heterozygosity by calculating the complement of HL (1-HL).

Birds used in the mate preference tests had heterozygosity levels of 0.52-1.00 with a mean of 0.81 ± 0.005 (mean ± SEM (standard error of the mean) (N=344). In the breeding season birds had heterozygosity levels between 0.52-1.00 with a mean of 0.83 ± 0.01 (adults: N=142; chicks: N=426).

<u>Relatedness</u>

We estimated marker-based relatedness by calculating the pairwise *r* following the method of Wang (2002) in the program Coancestry (Wang 2011). By calculating *r* for full sibling pairs (extra-pair chicks were excluded) using different methods we determined that, for this population and these microsatellite markers, the relatedness measure using the method of Wang (2002) best fitted our social pedigree (different methods of calculating pairwise r for full siblings and its associated standard error of the mean: 0.46 ± 0.004 SEM, Queller and Goodnight, 1989; 0.42 ± 0.07, Lynch and Ritland, 1999; 0.47 ± 0.004 Li et al., 1993; 0.48 ± 0.004, Wang, 2002). The relatedness values range from -1 to 1, in which values of 0 represent random allele sharing, and positive and negative values respectively represent more and less sharing than at random, based on the allele frequencies in the population. In the mate preference experiments the relatedness between the focal and stimulus birds, originating from different field sites, ranged between -0.31 and 0.405 and a mean of -0.01 ± 0.002 (N=2046). In the breeding season pairs within the experimental area had a relatedness of between -0.17 and 0.32 with a mean of 0.02 ± 0.01 (N=70).

C. Statistical analysis mate preferences (As described in the main paper, with more detail added)

To analyse the proportion of time that a focal bird spent associating with each stimulus bird we used a binomial generalized linear mixed model (GLMM) with a logit link function. The fixed part of the model contained as explanatory variables heterozygosity of both the focal and the stimulus birds, relatedness between each focal and stimulus dyad, offspring heterozygosity for each focal and stimulus dyad and sex of the focal bird. We also added the square of relatedness since a preference for moderately related individuals can be expected (Bateson 1983). To test for differences in preference depending on the chooser's traits we added the interaction between heterozygosity of the focal and the stimulus bird, and the interaction between the focal heterozygosity and relatedness. To test for sex differences, we also included interaction effects between sex and the previously mentioned explanatory variables.

Modelling the combined effect of continuous explanatory variables like the focal bird's heterozygosity, stimulus bird's heterozygosity and their interaction is necessarily sparse: only three parameters are used to describe the combined effect. To check whether the systematic trend captured in this way is not too restrictive, we also modelled the effect of these variables after categorization of each into three groups, based upon tertiles. Replacing the two regressors and their product by the categorized versions and their interaction, leads to a model with eight parameters replacing the earlier three. This model is more flexible than the original one, although it has its own shortcomings (Altman 2005).

For the random part of the GLMM we followed the experimental design as closely as possible, specifying the next random terms (on the logit scale): 1) random effects for stimulus birds, since each stimulus bird was tested repeatedly; 2) random slopes for focal birds with respect to the stimulus bird's heterozygosity, relatedness, and offspring heterozygosity, as each focal bird was tested multiple times. Together these random effects define the G-side covariance structure. Furthermore for the R-side covariance structure we allowed the six proportions per test to be negatively correlated (as they sum to one per six-choice test), by introducing a compound symmetric correlation structure at the proportion scale and we introduced an extra scale parameter, because we analysed a continuous proportion, for which the binomial

variance-mean relationship only holds up to a scale factor. The statistical analysis was performed using procedure PROC GLIMMIX from the SAS software system (version 9.3; SAS Institute Inc., Cary, NC). We fitted the model using backward elimination for the fixed part of the model, removing first higher order terms and later lower order terms if not significant ($P>0.01$). The reported P-value for an explanatory variable is the P-value in the last model in which it still occurred, or in the final model if not removed (see table 1).

Appendix S2: Offspring fledging weight minimal adequate model

Table S2 Offspring fledging weight minimal adequate model *All factors tested in the linear mixed model with the fledging weight (mg) of the offspring as the dependent variable (N=205). Biological (var ± sd: 1619 ± 40.24) and foster brood identity (15724 ± 125.40) were included as random factors (residual variation = 25030 ± 158.21). Given is the estimate, the degrees of freedom (df), the test statistic (t-value) and the significance (P-value). Using backwards elimination of factors, the P-values, df and test statistics given come from the last model in which the factor or interaction was included. Degrees of freedom for F- and t-tests were calculated using the degree of freedom approximation proposed by Kenward and Roger(1997).*

	**Estimate**	**df**	**Test statistic**	**P-value**
Minimal adequate model:				
Intercept	1585.82	54.80	31.06	<0.001
$HL_{foster\ male}$	535.65	29.13	2.24	*0.03*
Foster relatedness	-379.03	33.17	-1.18	0.25
Foster relatedness²	5655.18	34.91	3.19	**0.003**
Offspring sex (male)	-64.38	175.12	-2.67	**0.008**
Brood size	31.86	34.34	1.97	0.06
Catch date	7.13	28.24	0.13	0.90
Year (2015)	-100.83	28.61	-1.63	0.11
Year (2015) * catch date	34.59	28.65	0.55	0.59
Dropped terms:				
$HL_{offspring}$	11.29	120.82	0.06	0.95
$HL_{foster\ female}$	-103.86	29.69	-0.41	0.68
$HL_{foster\ female}$ * $HL_{foster\ male}$	1892.40	27.27	0.89	0.38
$HL_{foster\ female}$ * foster relatedness	-2380.31	29.04	-0.85	0.40
$HL_{foster\ male}$ * foster relatedness	143.28	29.02	0.06	0.96
$HL_{biological\ female}$	-218.12	18.84	-1.14	0.27
$HL_{biological\ male}$	-277.76	55.10	-1.34	0.19
$HL_{biological\ female}$ * $HL_{biological\ male}$	-1342.72	35.57	0.46	0.65
Biological relatedness	251.52	36.48	1.25	0.22
Biological relatedness²	-683.30	1274.16	0.54	0.60
$HL_{biological\ female}$ * biological relatedness	-2575.63	137.95	-1.40	0.16
$HL_{biological\ male}$ * biological relatedness	1181.14	39.36	-0.50	0.61

Appendix S3: Additional analyses

A. Observed and simulated distribution of relatedness and heterozygosity in pairs.

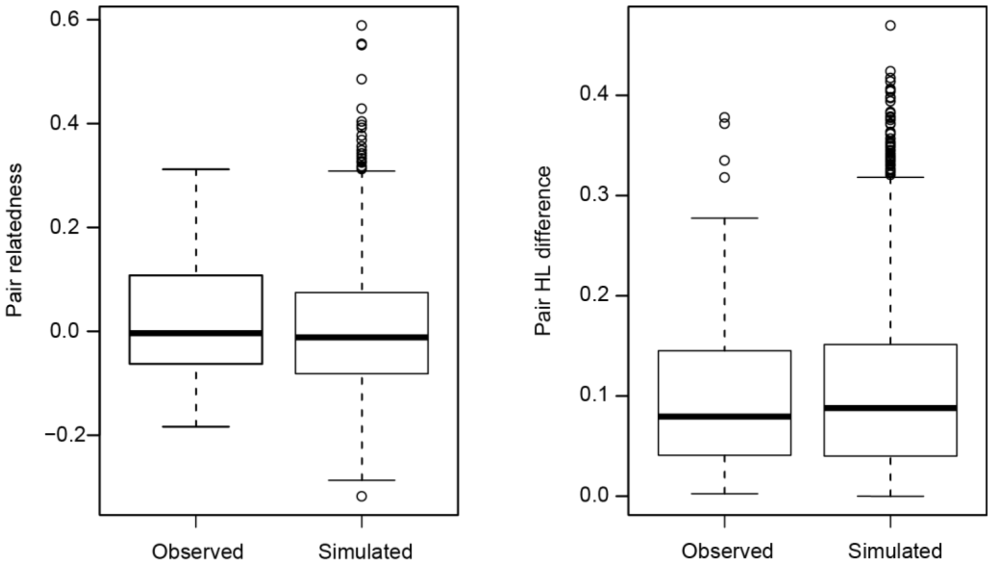

Figure S1. *Distributions of the observed versus the simulated pair relatedness and pair difference in heterozygosity.*

B. Accounting for spatial structure in mating patterns

Although the study site was relatively small, there is the possibility that individuals were constrained in their choice for a mate by the locally available potential mates. To check this we also compared the existing mating pattern to a different null model of random mating; one which considers the local mate availability. Using the 'nearest neighbour scenario' as used in Szulkin (2009), we paired the male and female of every pair to a known individual of the opposite sex breeding in the nest box nearest to the focal pair and compared the observed mating pattern with the simulated pattern. We tested for differences in the relatedness and heterozygosity similarity between the observed and simulated scenario of random mating using a non-parametric Wilcoxon matched-pair test. We found no differences between the observed and the simulated mating patterns, not for heterozygosity (WSR: females, T+=829, n1=n2=63, p=0.53; males, T+=969, n1=n2=63, p=0.87) or for relatedness (WSR: females, T+=3616, n1=n2=63, p=0.43; males, T+=900.5, n1=n2=63, p=0.91).

C. Accounting for effects of captures on mating patterns

By capturing and bringing the focal birds into captivity we may have unintentionally affected pair bonds and in extreme cases even split up pairs. To check whether testing the birds had any effect on mate choice we also compared mating patterns between untested pairs and pairs in which one or both were brought to the lab for testing. However, the mating patterns in these two groups did not differ. Both tested and untested birds did not mate differently from random mating in both heterozygosity and relatedness.

Heterozygosity (*Pairs of which one or both were tested*: random correlation 95% confidence interval = [-0.27, 0.28]; correlation breeding pairs = -0.12; N=52 breeding pairs; *Untested pairs*: random correlation 95% confidence interval = [-0.45, 0.48]; correlation breeding pairs = -0.36; N=18 breeding pairs. 10000 permutations).

Relatedness (*Pairs of which one or both were tested*: random relatedness confidence interval = [-0.035, 0.02]; average observed relatedness breeding pairs = 0.018; N=52 breeding pairs; *Untested pairs*: random relatedness confidence interval = [-0.020, 0.067]; average observed relatedness breeding pairs = 0.058; N=18 breeding pairs; 10000 iterations).

D. Alternative parametrization of mixed models

Results mate preference based on categorized heterozygosities

As described in Appendix S1-C we performed an extra analysis to check whether the specification of the interaction of continuous variables is not too restrictive, we also modelled the effect of these variables after categorization of each into three groups, based upon tertiles. First, the focal bird's and stimulus bird's heterozygosities were categorized into three groups based on tertiles. For the focal bird's heterozygosity we took the cut points -0.035767 and 0.064233. For the stimulus bird's heterozygosity we took the cut points -0.028627 and 0.051373. Next, in the GLMM we replaced the continuous heterozygosities and their interaction by main effects and interaction of the grouped heterozygosities. Results after removal of non-significant terms are given in table S3.

Table S3. Full model mate preference based on categorized heterozygosities. *Table consists of all factors tested in the binomial mixed model with proportion of time spent with each of the stimulus birds as the dependent variable (N_{focals}=116, N_{tests}= 359). Given is the degrees of freedom (numerator df and denominator df), the test statistic (F-value) and the significance (P-value).*

	Num DF	Den DF	F-Value	P-value
HL_{focal} (grouped)	2	252.70	1.54	0.22
$HL_{stimulus}$ (grouped)	2	142.10	0.71	0.49
HL_{focal} * $HL_{stimulus}$ (both grouped)	4	207.00	3.01	0.02
Relatedness	1	65.12	2.15	0.15
$Relatedness^2$	1	287.30	4.23	0.04
Relatedness*$HL_{stimulus}$	1	273.80	5.21	0.02

The estimated mean response (on the logit scale; mean ± se) for the combination of focal and stimulus heterozygosities, at the average value of relatedness (i.e. value zero for centered relatedness). We have also given the back transformed mean responses, which indicate fractions of time a focal bird spent on a specific stimulus bird. Without preference the fractions would be 1/6 = 0.167, as the focal bird can choose from six alternative stimulus birds.

Table S4. *Least square means for combinations of levels of grouped focal heterozygosity and grouped stimulus heterozygosity (± error) (**A**). Mean responses back transformed back to the probability (preference) scale for combinations of levels of grouped focal heterozygosity and grouped stimulus heterozygosity (**B**).*

A.

3	-1.84 ± 0.10	-1.77 ± 0.10	-1.65 ± 0.11
2	-1.68 ± 0.07	-1.56 ± 0.06	-1.60 ±0.07
1	-1.55 ± 0.08	-1.68 ± 0.08	-1.72 ± 0.09
	1	2	3

B.

3	0.14	0.15	0.16
2	0.16	0.15	0.17
1	0.18	0.16	0.15
	1	2	3

Results offspring fledging probability based on categorized heterozygosities

First, the foster mother, foster father and biological mother heterozygosities and biological relatedness were categorized into three groups based on tertiles. For the foster mother heterozygosity we took the cut points -0.02536803 and 0.03791166. For the foster father heterozygosity we took the cut points -0.03623277 and 0.06356268. For the biological mother heterozygosity we took the cut points -0.01245976 and 0.03742855. And for the biological relatedness we took the cut points -0.06911875 and 0.04628325. Next, in the GLMM we replaced the continuous heterozygosities and relatedness values and their interaction by main effects and interaction of the grouped heterozygosities and relatedness. Results after removal of non-significant terms are given in table S4.

Table S5. Full model offspring fledging probability based on categorized heterozygosities. *Table consists of all factors tested in the binary mixed model with the fledging probability of the offspring (0/1) as the dependent variable (N=272). Given is the degrees of freedom (df), the test statistic (χ^2 -value) and the significance (P-value).*

	**Df**	**χ^2**	**P-value**
$HL_{foster\ female}$ (grouped)	2	1.96	0.38
$HL_{foster\ male}$ (grouped)	2	2.11	0.35
$HL_{biological\ male}$	1	1.90	0.17
$HL_{biological\ female}$ (grouped)	2	0.91	0.64
Biological relatedness (grouped)	2	2.08	0.35
Offspring sex	1	0.85	0.36
Year	1	0.03	0.87
Hatch date	1	3.15	0.08
Brood size	1	1.64	0.2
$HL_{foster\ female}$ * $HL_{foster\ male}$ (both grouped)	4	18.97	**0.0008**
$HL_{biological\ female}$ * biological relatedness (both grouped)	4	13.12	0.01

The estimated mean response (on the logit scale; mean ± se) for the combination of foster mother and foster father heterozygosities. We have also given the back transformed mean responses, which indicate the fledging probability for the combination of foster mother and foster father heterozygosity.

Table S6. *Least square means for combinations of levels of grouped foster mother and foster father heterozygosities (± error) (A). Mean responses back transformed back to the probability (of fledging) scale for combinations of levels of grouped foster mother and foster father heterozygosities (B).*

A.

3	-0.73 ± 1.25	1.60 ± 0.98	1.64 ± 1.60
2	-0.58 ± 0.98	0.80 ± 1.13	1.99 ± 1.11
1	1.66 ± 1.09	0.91 ± 1.19	-3.51 ± 1.09
	1	2	3

B.

3	0.33	0.83	0.84
2	0.36	0.69	0.88
1	0.84	0.71	0.03
	1	2	3

The estimated mean response (on the logit scale; mean ± se) for the combination of biological mother heterozygosity and her relatedness with the biological father. We also give the back transformed mean responses, which indicate the fledging probability for the combination of foster mother and foster father heterozygosity.

Table S7. *Least square means for combinations of levels of grouped biological mother heterozygosity and the grouped relatedness with the biological father (± error) (**A**). Mean responses back transformed back to the probability (of fledging) scale for combinations of levels of grouped biological mother heterozygosity and the grouped relatedness with the biological father*

B

3	2.49 ± 1.24	-0.33 ± 1.00	-0.84 ± 1.05
2	1.27 ± 0.97	1.60 ± 1.01	-0.16 ± 0.93
1	-1.46 ± 0.96	-0.02 ± 1.01	1.25 ± 1.04
	1	2	3

3	0.92	0.42	0.30
2	0.78	0.83	0.46
1	0.19	0.49	0.78
	1	2	3

Appendix S4: Additional results with weak significance

A. Mate preferences

Both males and females tended to spend more time with moderately related individuals than with very related or unrelated individuals (relatedness^2, GLMM: $F_{1,274.2}$=-2.39, p=0.02). This effect was also influenced by the heterozygosity of the stimulus bird (Fig. 2b; relatedness* $HL_{stimulus}$, GLMM: $F_{1,277,1}$=-2.06, p=0.04). Individuals tended to spend more time when the stimulus bird was heterozygous and relatively unrelated or when the stimulus bird was homozygous and relatively related.

Individuals tended to prefer a moderately related mate rather than a genetically dissimilar mate with whom they could increase offspring heterozygosity (see also Kleven *et al.* 2005; Oh & Badyaev 2006). It has been suggested that such patterns can occur when individuals balance any potential costs and benefits of in- and outbreeding by finding a mate with the 'optimal' genetic similarity, (Neff 2004; Greeff *et al.* 2009; Richard *et al.* 2009). We did not find any fitness effects of this preference. These genetic effects may be very small, or only appear later in life, and because of this we did not see them reflected in the reproductive success. Although we did find a rearing effect of parental relatedness on chick weight, this effect was the inverse of the preference tendency. Possibly this preference was a genetic preference for indirect benefits or optimal outbreeding (Bateson 1983), despite the negative direct effects of this preference on chick weight.

B. Reproductive success

Heterozygous foster fathers tended to raise heavier offspring (Fig. 3a; $HL_{foster\ male}$, LMM: $t_{29.13}$=2.24, P=0.03). Biological fathers (within-pair or extra-pair sire), which were more homozygous tended to produce offspring with a higher fledging probability (Fig. 4a; $HL_{biological\ male}$, GLMM: Z=-2.07, P=0.04).

Heterozygous parents have previously been shown to invest more in their offspring (Foerster *et al.* 2003; García-navas *et al.* 2009). Heterozygous blue tits for instance provisioned more than homozygous individuals (García-navas *et al.* 2009). Here we found that the absolute

heterozygosity levels of foster fathers tended to be correlated with a higher fledging weight, which indeed suggests that these males may be able to invest more resources in their offspring and give direct reproductive benefits (García-navas *et al.* 2009). Females on the other hand often provision more than males and are often more responsive to male provisioning levels and chick begging. Because of this we may not see the same correlation with heterozygosity as in males. Conversely, the heterozygosity levels of the biological fathers tended to have a negative effect on fledging success. Possibly homozygous birds and offspring are better locally adapted to the environment and heterozygous males were more likely to sire offspring that is less adapted. Apart from genetic effects the higher fledging success might also work through other pre-hatching effects that may be related to male attractiveness, such as maternal investment in egg size (Cunningham & Russell 2000; Horváthová *et al.* 2012), yolk carotenoids (Marri & Richner 2014) or yolk androgens (Gil *et al.* 1999; Kingma *et al.* 2009)

Appendix S5 Full statistical models

Table S8 – Mate preferences full model. *Table consists of all factors tested in the binomial mixed model with proportion of time spent with each of the stimulus birds as the dependent variable (N_{focals}=116, N_{tests}= 359). Given is the degrees of freedom (df), the test statistic (F-value) and the significance (P-value). A random effect for stimulus bird identity (mean ± SE; 0.23 ± 0.05) and random slopes for focal bird identity with respect to the stimulus birds heterozygosity (2.37 ± 0.76), relatedness (1.31 ± 0.51), and offspring heterozygosity (3.21 ± 2.09), and a random effect for test number (to allow for negative correlations among association times within one six-choice test; (-13.60 ± 0.51) and an extra scale parameter on the original scale, were included in the model. Degrees of freedom for F-tests were calculated using the degree of freedom approximation proposed by Kenward and Roger(1997).*

	**Num df**	**Denom df**	**Test statistic**	**P-value**
HL_{focal}	1	191.50	2.76	0.10
$HL_{stimulus}$	1	164.40	0.42	0.52
HL_{focal} * $HL_{stimulus}$	1	62.56	6.47	**0.01**
Relatedness	1	124.40	2.37	0.13
Relatedness^2	1	293.00	3.29	0.07
Relatedness * $HL_{stimulus}$	1	278.50	4.19	**0.04**
$HL_{offspring}$	1	69.07	0.00	0.99
Sex (female)	1	75.29	0.00	0.98
$HL_{stimulus}$ * sex (female)	1	164.40	1.22	0.27
HL_{focal} * $HL_{stimulus}$ * sex (female)	1	61.55	1.11	0.30
Relatedness * sex (female)	1	108.10	0.93	0.34
Relatedness^2 * sex (female)	1	293.80	0.37	0.54
$HL_{offspring}$ * sex (female)	1	45.21	0.79	0.38

Table S9 - Offspring fledging probability full model. *Table consists of all factors tested in the binary mixed model with the fledging probability of the offspring (0/1) as the dependent variable (N=272). Random effects for biological brood (var ± sd: 0.00 ± 0.00) and foster brood (3.72 ± 1.93) were included in the model. Given is the test statistic (Z-value) and the significance (P-value). Biological and foster brood identity were included as random factors.*

	**Test statistic**	**P value**
Intercept	0.49	0.62
$HL_{offspring}$	0.30	0.76
$HL_{foster\ female}$	-1.71	0.09
$HL_{foster\ male}$	1.09	0.28
$HL_{foster\ female}$ * $HL_{foster\ male}$	3.42	**<0.001**
Foster relatedness	1.44	0.15
$HL_{foster\ female}$ * Foster relatedness	0.80	0.43
$HL_{foster\ male}$ * Foster relatedness	0.38	0.70
$HL_{biological\ female}$	-1.99	*0.047*
$HL_{biological\ male}$	-1.44	0.15
$HL_{biological\ female}$ * $HL_{biological\ male}$	-0.08	0.93
Biological relatedness	2.10	*0.036*
$HL_{biological\ female}$ * biological relatedness	-3.06	**<0.01**
$HL_{biological\ male}$ * biological relatedness	-0.73	0.47
Offspring sex (male)	-0.67	0.51
Brood size	-0.99	0.32
Hatch date	1.07	0.28
Year (2015)	0.10	0.92

Table S10 – Offspring fledging weight full model. *All factors tested in the linear mixed model with the fledging weight (mg) of the offspring as the dependent variable (N=205). Biological (var ± sd: 0.00 ± 0.00) and foster brood identity (17943 ± 134.0) were included as random factors (residual variation = 28767 ± 169.6). Given is the estimate, the degrees of freedom (df), the test statistic (t-value) and the significance (P-value). Given are the P-values, df and test statistics. Degrees of freedom for F- and t-tests were calculated using the degree of freedom approximation proposed by Kenward and Roger (1997).*

	df	Test statistic	P-value
Intercept	30.78	11.47	<0.001
$HL_{offspring}$	118.02	-0.04	0.97
$HL_{foster\ female}$	21.45	-0.64	0.53
$HL_{foster\ male}$	19.52	1.46	0.16
$HL_{foster\ female}$ * $HL_{foster\ male}$	20.17	1.15	0.27
Foster relatedness	23.23	-1.48	0.15
Foster relatedness^2	22.04	2.81	**0.01**
$HL_{foster\ female}$ * foster relatedness	24.84	-0.66	0.51
$HL_{foster\ male}$ * foster relatedness	22.42	0.86	0.40
$HL_{biological\ female}$	97.22	-0.44	0.66
$HL_{biological\ male}$	109.05	-1.30	0.20
$HL_{biological\ female}$ * $HL_{biological\ male}$	48.27	0.14	0.89
Biological relatedness	109.19	1.94	0.05
Biological relatedness^2	134.51	-1.56	0.12
$HL_{biological\ female}$ * biological relatedness	124.03	-1.99	0.05
$HL_{biological\ male}$ * biological relatedness	96.70	-1.26	0.21
Offspring sex (male)	123.90	-2.42	**0.02**
Brood size	30.77	1.39	0.18
Catch date	31.16	1.23	0.23
Year (2015)	30.50	0.14	0.89
Year (2015) * catch date	31.58	-1.06	0.30

References

1.Altman, D.G. (2005). Categorizing Continuous Variables. In: *Encyclopedia of Biostatistics* (eds. Armitage, P. & Colton, T.). John Wiley & Sons, Ltd., Chichester, England

2.Aparicio, J.M., Ortego, J. & Cordero, P.J. (2006). What should we weigh to estimate heterozygosity, alleles or loci? *Mol. Ecol.*, 15, 4659–4665

3.Balloux, F., Amos, W. & Coulson, T. (2004). Does heterozygosity estimate inbreeding in real populations? *Mol. Ecol.*, 13, 3021–3031

4.Bateson, P. (1983). Optimal outbreeding. In: *Mate choice* (ed. Bateson, P.). Cambridge University Press, Cambridge, pp. 257–277

5.Cunningham, E.J. & Russell, A.F. (2000). Egg investment is influenced by male attractiveness in the mallard. *Nature*, 404, 74–7

6.David, P., Pujol, B., Viard, F., Castella, V. & Goudet, J. (2007). Reliable selfing rate estimates from imperfect population genetic data. *Mol. Ecol.*, 16, 2474–2487

7.Foerster, K., Delhey, K., Johnsen, A., Lifjeld, J.T. & Kempenaers, B. (2003). Females increase offspring heterozygosity and fitness through extra-pair matings. *Nature*, 425, 714–717

8.García-navas, V., Ortego, J. & Sanz, J.J. (2009). Heterozygosity-based assortative mating in blue tits (Cyanistes caeruleus): implications for the evolution of mate choice. *Proc. R. Soc. B Biol. Sci.*, 276, 2931–40

9.Gil, D., Graves, J., Hazon, N. & Wells, A. (1999). Male Attractiveness and Differential Testosterone Investment in Zebra Finch Eggs. *Science (80-.).*, 286, 126–129

10.Greeff, J.M., Jansen van Vuuren, G.J., Kryger, P. & Moore, J.C. (2009). Outbreeding and possibly inbreeding depression in a pollinating fig wasp with a mixed mating system. *Heredity (Edinb).*, 102, 349–356

11.Horváthová, T., Nakagawa, S. & Uller, T. (2012). Strategic female reproductive investment in response to male attractiveness in birds. *Proc. R. Soc. B Biol. Sci.*, 279, 163–70

12.Kawano, K.M. (2003). Isolation of polymorphic microsatellite markers in the great tit (Parus major minor). *Mol. Ecol. Notes*, 3, 314–315

13.Kenward, M.G. & Roger, J.H. (1997). Small Sample Inference for Fixed Effects from Restricted Maximum Likelihood. *Biometrics*, 53, 983–997

14.Kingma, S.A., Komdeur, J., Vedder, O., Von Engelhardt, N., Korsten, P. & Groothuis, T.G.G. (2009). Manipulation of male attractiveness induces rapid changes in avian maternal yolk androgen deposition. *Behav. Ecol.*, 20, 172–179

15.Kleven, O., Jacobsen, F., Robertson, R.J. & Lifjeld, J.T. (2005). Extrapair mating between relatives in the barn swallow: a role for kin selection? *Biol. Lett.*, 1, 389–92

16.Li, C.C., Weeks, D.E. & Chakravarti, A. (1993). Similarity of DNA fingerprints due to chance and relatedness. *Hum. Hered.*, 43, 45–52

17.Lynch, M. & Ritland, K. (1999). Estimation of pairwise relatedness with molecular markers. *Genetics*, 152, 1753–1766

18.Marri, V. & Richner, H. (2014). Yolk carotenoids increase fledging success in great tit nestlings. *Oecologia*

19.Marshall, T.C., Slate, J., Kruuk, L.E.B. & Pemberton, J.M. (1998). Statistical confidence for likelihood-based paternity. *Mol. Ecol.*, 639–655

20.Neff, B.D. (2004). Stabilizing selection on genomic divergence in a wild fish population. *Proc. Natl. Acad. Sci. U. S. A.*, 101, 2381–2385

21.Oh, K.P. & Badyaev, A. V. (2006). Adaptive genetic complementarity in mate choice coexists with selection for elaborate sexual traits. *Proc. R. Soc. B Biol. Sci.*, 273, 1913–1919

22.Queller, D.C. & Goodnight, K.F. (1989). Estimating relatedness using genetic markers evolution. *Evolution (N. Y).*, 43, 258–275

23.Richard, M., Losdat, S., Lecomte, J., de Fraipont, M. & Clobert, J. (2009). Optimal level of inbreeding in the common lizard. *Proc. R. Soc. B Biol. Sci.*, 276, 2779–2786

24.Saladin, V., Bonfils, D., Binz, T. & Richner, H. (2003). Isolation and characterization of 16 microsatellite loci in the Great Tit Parus major. *Mol. Ecol. Notes*, 3, 520–522

25.Saladin, V. & Richner, H. (2012). A set of 48 microsatellite loci for the great tit Parus major including 15 novel markers. *Mol. Ecol. Resour.*, 12, 185–189

26.Stoffel, M.A., Esser, M., Kardos, M., Humble, E., Nichols, H., David, P., *et al.* (2016). inbreedR: an R package for the analysis of inbreeding based on genetic markers. *Methods Ecol. Evol.*, 7, 1331–1339

27.Szulkin, M., Zelazowski, P., Nicholson, G. & Sheldon, B.C. (2009). Inbreeding avoidance under different null models of random mating in the great tit. *J. Anim. Ecol.*, 78, 778–788

28.Wang, J. (2002). An estimator for pairwise relatedness using molecular markers. *Genetics*, 160, 1203–1215

29.Wang, J. (2011). Coancestry: A program for simulating, estimating and analysing relatedness and inbreeding coefficients. *Mol. Ecol. Resour.*, 11, 141–145

30.Wang, M.T., Hsu, Y.C., Yao, C. Te & Li, S.H. (2005). Isolation and characterization of 12 tetranucleotide repeat microsatellite loci from the green-backed tit (Parus monticolus). *Mol. Ecol. Notes*, 5, 439–442
